# Drug discovery in ophthalmology: past success, present challenges, and future opportunities

**DOI:** 10.1186/s12886-016-0188-2

**Published:** 2016-01-16

**Authors:** Nicholas J. D. Gower, Robert J. Barry, Matthew R. Edmunds, Lucy C. Titcomb, Alastair K. Denniston

**Affiliations:** Queen Elizabeth Hospital Birmingham, University Hospitals Birmingham NHS Foundation Trust, Birmingham, UK; Institute of Inflammation and Ageing, College of Medical and Dental Sciences, University of Birmingham, Edgbaston, Birmingham, UK; Birmingham and Midland Eye Centre, Sandwell & West Birmingham Hospitals NHS Trust, Birmingham, UK

**Keywords:** Ophthalmology, Drug discovery, Pharmaceutical

## Abstract

**Background:**

Drug discovery has undergone major transformations in the last century, progressing from the recognition and refinement of natural products with therapeutic benefit, to the systematic screening of molecular libraries on whole organisms or cell lines and more recently to a more target-based approach driven by greater knowledge of the physiological and pathological pathways involved. Despite this evolution increasing challenges within the drug discovery industry are causing escalating rates of failure of development pipelines.

**Discussion:**

We review the challenges facing the drug discovery industry, and discuss what attempts are being made to increase the productivity of drug development, including a refocusing on the study of the basic biology of the disease, and an embracing of the concept of ‘translational research’. We consider what ophthalmic drug discovery can learn from the sector in general and discuss strategies to overcome the present limitations. This includes advances in the understanding of the pathogenesis of disease; improvements in animal models of human disease; improvements in ophthalmic drug delivery and attempts at patient stratification within clinical trials.

**Summary:**

As we look to the future, we argue that investment in ophthalmic drug development must continue to cover the whole translational spectrum (from ‘bench to bedside and back again’) with recognition that both biological discovery and clinical understanding will drive drug discovery, providing safe and effective therapies for ocular disease.

## Background

The identification of effective and safe therapies that ameliorate disease is central to the practice and progress of medicine. Drug discovery has undergone major transformations in the last century, progressing from the recognition and refinement of natural products with therapeutic benefit (such as the use of cardiac glycosides extracted from plants of the genus Digitalis), to the systematic screening of molecular libraries on whole organisms or cell lines and more recently to a more target-based approach driven by greater knowledge of the physiological and pathological pathways involved.

Major success stories such as anti-vascular endothelial growth factor (VEGF) therapies must be seen in the context of the very great challenges currently facing those involved in drug discovery and development, both in ophthalmology and in the sector as a whole. Hay et al. noted that looking at the 2003–2011 data across all specialities the number of new drugs approved by the US Food and Drug Administration (FDA) has actually fallen despite a 62 % increase in the number of compounds in development, and a doubling of R&D expenditure over the last decade [1–3]. The authors noted that in this period an average of 26 new drugs (either new molecular entities, NME, or biological products licensed through a Biological Licence Application (BLA)) were approved per year, a 25 % decline on the average rates of approval in the 1990s [4].

In this review we consider the changing landscape of drug discovery. We will start by looking back at the history of the anti-VEGF drug discovery programme, and then consider the challenges facing both the drug discovery industry as a whole and more specifically the ophthalmic drug discovery sector. Finally, we will look to the future of drug discovery in light of these challenges and consider what ophthalmic drug discovery can learn from the sector in general and discuss strategies to overcome the present limitations. As we look to the future, we argue that investment in ophthalmic drug development must continue to cover the whole translational spectrum (from ‘bench to bedside and back again’) with recognition that both biological discovery and clinical understanding will drive drug discovery, providing safe and effective therapies for ocular disease.

## Discussion

### Past success: VEGF from basic science to the bedside

It is over 70 years since the importance of the development of neovascular supply to tumour growth was first demonstrated [5]. Forty years later, following the cloning of VEGF as an angiogenic enhancing factor, research using pharmacological and genetic tools resulted in the clinical development of bevacizumab, a VEGF specific antibody, which has today been approved for therapy of multiple tumour types [6–10].

The first indicators that this might be relevant to the field of ophthalmology were present as early as the 1940s when it was proposed that a diffusible factor responsible for the development of the normal retinal vasculature and for pathological neovascularization in proliferative diabetic retinopathy [11]. By the 1990s VEGF had been identified as a potential mediator of intraocular neovascularization and could be found in choroidal neovascular membranes from individuals with wet age related macular degeneration (AMD) [12, 13]. Proof-of-concept studies showed that VEGF blockade resulted in inhibition of intraocular neovascularization in a variety of animal models [14–16]. In 2004 the results of the first phase III clinical trial using an anti-VEGF to treat neovascular AMD was published, demonstrating that intravitreal administration of the aptamer pegaptanib sodium (later launched as Macugen), reduced visual loss [17].

Concurrently ranibizumab, a Fab fragment of the humanised anti-VEGF bevacizumab, was being developed [8]. Ranibizumab had the theoretical advantage over pegaptanib sodium that it bound more active isoforms of VEGF, and the advantage over bevacizumab that it was smaller and theoretically might have better penetration through the retina [18]. Prior to the first human studies it was tested in a primate model of laser-induced choroidal neovascularisation (CNV), showing resolution of CNV on fluorescein angiography [19]. The first phase I study identified the maximum tolerated intravitreal dose (of 500 micrograms), with higher doses resulting in significant intraocular inflammation. Arising from this a phase I/II study proceeded to test safety and efficacy of monthly injections of either 300 or 500 micrograms intravitreal ranibizumab. This was a larger study of 64 patients, with an open label randomised design vs usual care [20]. Based on these positive results, the phase III study, MARINA, was undertaken which was a multicentre, two-year, double-blind, sham-controlled study of monthly intravitreal ranibizumab (either 300 micrograms or 500 micrograms or sham injections). This study of 716 patients reached its primary endpoint, notably that the percentage of patients losing fewer than 15 letters visual acuity at 12 months was 94.5 % and 94.6 % for ranibizumab (0.3 mg and 0.5 mg dose respectively) vs 62.2 % for sham injections (P < 0.001 for both) [21].

It is hard to over-estimate the impact of these studies in the field of Medical Retina. Before anti-VEGF therapy, the main therapeutic options for patients with wet AMD were laser photocoagulation, surgical resection of CNV, or, in the latter years, photodynamic therapy, all of which had limited efficacy, could be used only in selected cases and were sometimes associated with significant ocular morbidity [22]. The advent of anti-VEGF drugs has revolutionised not only wet AMD but the treatment of a range of retinovascular diseases, with profound benefit to patients with these conditions [23].

Understanding why the drug development programme targeting VEGF was so successful compared to many notable failures in the industry is difficult. The drugs were the product of decades of research and were thus underpinned with extensive knowledge of the basic pathophysiology of the disease area. The programme also built on advances in the design of biological based therapies to develop variants like ranibizumab for the treatment of wet AMD. One can speculate about which of these unique features led to success, however the fact remains that despite this success the drug discovery industry is today facing unprecedented challenges. This review will now look at these challenges in more detail and also consider those specific to ocular drug discovery.

### Present challenges

#### Challenges facing the drug discovery industry generally

A Forbes analysis in 2013 estimated that the larger pharmaceutical companies were now spending around $5 billion per drug approved [24]. A recent review looking at drug approval rates from 2003 to 2011 which included 835 drug developers (including big pharma, small biotechs, and specialty companies) found that only 15 % of drugs made it from phase I to approval based on their lead indication; when looking at ‘all indications’ the success rate was only 10 %. Failure of drug candidates occurred all the way along the pipeline with rates of around 35 % at phase I, 68 % at phase II and 30-40 % at phase III [1]. At this level of innovation the industry has failed to develop adequate numbers of new drugs to replace existing treatments that are at the end of their patent life [25, 26]. Within the industry there is significant debate about the causes and consequences of this innovation drought [27].

Many industry reports have identified drug efficacy as a major factor in the increase in drug failure rates [28]. Since efficacy is a fundamental component of the early drug discovery process it would suggest that a key part of the productivity crisis is how the pre-clinical drug discovery process is performed and how specific drugs are selected to continue into clinical development. In particular target selection has been highlighted as one of the most important determinants of attrition and overall R&D productivity [25, 29].

Target-based screening and phenotypic screening are the two most commonly used preclinical strategies to identify potential new drug candidates [29]. Phenotypic screening simply looks at the effect that a particular compound has on the cell, tissue or whole organism and so does not require a detailed understanding of the target or mechanism of action. In contrast, target-based screening seeks to identify a biologically relevant target first, and then develops drugs that achieve a desired modification of that target. Historically drug development has been dependent on phenotypic screening, but since the 1990s the target-based approach has become increasingly common to the point that it rapidly became the ‘preferred’ method of drug discovery. Both techniques have their strengths, and interestingly, data for FDA approvals for NME and biological products from 1999 to 2008 suggest that target-based approach is less likely to result in regulatory approval of a drug compared to projects based on a phenotypic approach [29, 30]. It is likely however that it is not that the target-based technique is flawed, but rather that identifying the target is only one part of the process of using a directed approach to developing a successful drug. It may be argued that we need even greater depth of biological understanding and appreciation of the molecular mechanism of action of the proposed drug (in contrast to the relatively ‘blind’ approach of phenotypic screening) to fully realise the benefits of the target-based approach. The need for a better understanding of the pathophysiology, clinical presentation and course of disease is widely acknowledged [28]. In addition, a good knowledge of the limitations of current therapies is crucial to understand the requirements of possible future treatments.

#### Opportunities and challenges in ocular drug discovery

Despite highlights like the anti-VEGF story, there has been a chronic lack of innovation in ocular drug development despite an expanding market potential [31]. This may, in part, be down to the unique features of the eye which can both enhance and impede ocular drug discovery and drug delivery. A major advantage of ocular disease is that in contrast to other parts of the central nervous system, drug delivery can be more targeted using either drops applied to the surface of the eye or injections made directly into the eye [32]. These routes of drug delivery minimise systemic toxic effects and therefore enhance therapeutic indices. However, some anatomical and physiological features of the eye represent challenges to drug discovery. In particular ocular barriers including tear dilution, blood flow, lymphatic clearance and blood-ocular barriers impede drug transport and lower the efficacy of many drugs [33, 34]. Consideration also needs to be given to ‘real world’ factors such as patient adherence and, for self-administered therapies such as eye drops, patient technique [35–37].

Outcome measures are a key area for ophthalmic studies, and have a number of distinct features in ocular disease. On the plus side, a number of key outcome measures are non-invasive (such as visual acuity, intraocular pressure, anterior chamber cell count) enabling good patient acceptability to frequent monitoring and early detection of responses for some conditions. The problem is that most outcome measures in ophthalmology are subjective whether reported by the patient (e.g. visual symptoms) or the clinician (e.g. anterior chamber cells), many are imprecise and many are not quantitative (or are only semi-quantitative). Indeed most measures of inflammation (such as the National Eye Institute (NEI) vitreous haze score favoured for most studies of posterior segment-involving uveitis) have the dubious honour of being subjective, semi-quantitative, imprecise measurements with only moderate inter-observer agreement [38, 39].

Another feature of ophthalmic disease is how the prevalence of many of its major causes of disability (such as AMD or glaucoma) increases with age. In the context of our ageing populations, this is variously viewed as a ‘timebomb’ or an ‘opportunity’; it certainly is an important motivation for further innovation within ophthalmic drug discovery [40]. One metric for analysing progress in ocular drug discovery is to look at the growth trends in the global ophthalmic therapeutic market. Data suggests that this market is growing at two and a half times the rate of the overall pharmaceutical industry [41]. Predictions suggest it will have continued to grow and reached $20.6 billion by the end of 2014. Therefore, many commercial opportunities for ophthalmic drugs exist, with high revenues possible within the next decade.

It is interesting to note that, despite glaucoma being a dominant force in the ophthalmic market, at the time of writing no new classes of glaucoma drug have been successfully launched since Xalatan (latanoprost) in 1996 [42]. This lack of innovation has caused the glaucoma market to be subject to increasingly major revenue depletion due to recent and forthcoming patent expiries. Expiry with a failure to launch new proprietary drugs means that generics are predicted to make a significant impact on valuations of the glaucoma therapeutics market over the near term. This is well illustrated by Pfizer’s estimate that in the UK National Health Service spending on Xalatan fell by £16 m (from £52 m to £36 m) a year from 2012 following patent expiry. Interestingly Lumigan (bimatoprost) had been expected to come off patent in 2014, however Allergan has introduced a novel formulation with a 0.01 % concentration for which it has been successful in obtaining a US patent extending to 2025 (Patent US8933127 B2). Until recently major growth areas in the glaucoma market were around ‘fine-tuning’ existing products, notably an emphasis on preservative-free preparations and combination therapies rather than the development of new classes of drugs.

Despite the unique challenges posed in ocular drug discovery and the historical lack of innovation things appear to be changing. By analysing a number of the drugs currently undergoing late stage clinical trials in glaucoma we see products from both established pharmaceutical companies and specialist biotechnology firms (Table 1), many of which are entering the ophthalmology market for the first time, tempted by lower barriers to entry and large market sizes [31]. Over the past decade there have been considerable advances in the understanding of the pathogenesis of ocular diseases, including glaucoma, and an improved knowledge of the ability of the eye to respond to physical and pharmacologic intervention. These advances are seen in the clinical pipeline for glaucoma with the development of novel groups of drugs such as nitric oxide-donating prostaglandin F2-alpha analogues such as Vesneo (latanoprostene bunod) and Rho kinase (ROCK) inhibitors such as AR-12286, and combination molecules based on this compound such as Rhopressa (Aerie Pharmaceuticals) which is now in Phase III trials [42, 43]. A number of these drugs are also the products of new treatment modalities (such as RNA interference technologies [44]) and innovations in improving delivery (such as punctal plug technologies [45]). These new treatments aim to improve outcomes for patients whilst reducing the side-effects that limit current treatments.

**Table 1:** A selected list of glaucoma drugs in late-stage clinical trails

Drug name	Company	Mechanism of action	Clinical phase	Clinical trial identifier	Ref
Vesneo	Bausch & Lomb and Nicox	Nitric-oxide-donating prostaglandin F2a analogue	Phase III	NCT01749930 NCT01895972 NCT01749904	[43]
Trabodenoson	Inotek Pharmaceuticals	Adenosine receptor agonist	Entering Phase III in end 2015	NCT02565173	[103]
Rhopressa	Aerie Pharmaceuticals	Inhibits Rho Kinase (ROCK), and the norepinephrine transporter (NET)	Phase III	NCT02246764 NCT02207621 NCT02207491 NCT02558374	[104]
Rocla tan	Aerie Pharmaceuticals	Combination of Rhopressa and latanoprost	Phase III	NCT02558400	[105]
Bamosiran	Sylentis	Small interference RNA (siRNA) inhibitor of beta 2 adrenergic receptor	Phase II	NCT02250612	[44]
Latanoprost punctal plug delivery system (L-PPDS)	Mati Therapeutics Inc	Sustained delivery of latanoprost via a punctal plug	Phase II	NCT02014142 NCT01481051 NCT01481077 NCT00820300 NCT00845299 NCT01229982 NCT00821002 NCT00967811 NCT 01037036	[45]

Although these new drugs have huge potential, even those at a late stage of clinical development face stringent regulatory hurdles and subsequently a high rate of failure. Overall, however, the future holds enormous opportunity for innovative ophthalmic drug discovery and development.

### The future in ocular drug discovery

As highlighted earlier, it is suggested that a key cause of the productivity crisis affecting the drug discovery industry is at the level of early-stage drug discovery, particularly around target selection. Improved understanding of pathogenesis, better disease modelling, more effective drug delivery techniques and appropriate patient stratification for clinical trials are all proposed to reverse the dwindling success rates of the last few years.

#### Understanding the pathophysiology of a disease

Just as the understanding of VEGF unlocked an effective therapy for wet AMD, so the better understanding of the pathophysiology of various ocular inflammatory diseases is now opening them up to more targeted therapies, which have the potential to be effective and safer than current, usually corticosteroid-based, regimens.

Uveitis describes a heterogeneous group of disorders characterised by intraocular inflammation. There are currently no non-corticosteroid treatments licensed by the FDA for the treatment of uveitis, despite a recognition that this is an area of high unmet need. Although uveitis specialists use a number of standard immunosuppressants off-label (e.g. methotrexate, mycophenolate, etc.), animal models of uveitis and in vitro human data are enabling the identification of specific pathways that may be targeted in uveitis, including the role of interleukin-17 (IL-17) secreting T- helper cells (Th17), and the pro-inflammatory cytokines IL-6 and IL-1β.

Over the last decade IL-17A has become recognised as a key mediator in a range of immune-mediated conditions including inflammatory bowel disease, rheumatoid arthritis and multiple sclerosis. Studies in patients with active uveitis identified higher levels of the cytokine in the peripheral blood compared to healthy controls or patients with inactive disease. In 2007 Amadi-Obi et al. showed that IL17A was elevated in an animal model of experimental autoimmune uveoretinitis and was found to upregulate IL-12 production in retinal cells [46]. They also noted that IL-17A inhibition reduced disease activity in the animal uveitis model. The development of secukinumab (AIN457, Novartis Pharmaceuticals Corporation), a selective high-affinity fully human monoclonal antibody for IL-17A, showed promise in a proof-of-concept trial, encouraging a rapid translation to phase III studies. Disappointingly these phase III studies failed to demonstrate the beneficial effect seen in the earlier studies, possibly because they used a subcutaneous form with lower bioavailability than the original intravenous preparation [47]. In light of this observation the sponsor (Novartis Pharmaceuticals) is now undertaking further assessment of the intravenous preparation. A recent dose-ranging phase II trial of intravenous (IV) vs subcutaneous (SC) secukinumab has shown responses of 62 % and 73 % for the IV dose (fortnightly 10 mg/kg and 30 mg/kg respectively) compared to 33 % for SC dose (300 mg fortnightly). The intravenous form of secukinumab was well tolerated and thus it would appear that the intravenous form would be a good candidate for phase III studies [48].

Other key inflammatory cytokines identified to have a central role in both in vitro and animal studies are IL-6 and IL1β. IL-6 is found at elevated levels in the aqueous humour of patients with active uveitis, and has been shown to be upregulated early in the disease process [49–51]. Tocilizumab (Actemra, Genentech, Inc.) is a humanized monoclonal IL-6 receptor antibody which inhibits downstream signalling [52]. Tocilizumab has an established role in the treatment of RA, and there is now emerging data based on case series to support its use in refractory uveitis [53–56] and uveitic macular oedema [57–59]. There are two phase I/II clinical trials of tocilizumab currently underway: one for non-infectious intermediate, posterior or panuveitis (the STOP-UVEITIS study; NCT01717170) and one specifically on uveitis associated with JIA (NCT01603355).

Another example of an ophthalmic disease in which laboratory research of human biofluids and tissues has identified a candidate target molecule for treatment is Thyroid Eye Disease (TED). Autoantibodies to insulin-like growth factor-1 receptor (IGF-1R) are thought to be involved in the pathogenesis of this inflammatory condition of the structures of the eye socket, particularly the extraocular muscles and orbital adipose tissue. Indeed, previous studies have demonstrated that IGF-1R expression is higher on orbital fibroblasts, as well as T and B lymphocytes from TED patients as compared to healthy controls [60–62]. Furthermore, serum from patients with TED triggers orbital fibroblasts to produce T cell chemoattractants and inflammatory mediators, effects abrogated by IGF-1R monoclonal antibody [63, 64]. Finally, microarray analysis identifies IGF-1R signalling gene overexpression in orbital tissue from patients with TED [65]. As a result Teprotumumab, a human monoclonal antibody that targets IGF-1R, and which was originally designed for the treatment of solid and hematologic tumors, is now undergoing trials in TED [66].

#### Improvements in modelling human disease

The lack of availability of predictive animal models limits the ability to study human disease and to select therapeutics that might succeed or fail during clinical investigation. By developing preclinical animal models that more accurately recapitulate human disease many industry insiders believe researchers will be better placed to reliably test drug efficacy, model therapeutic mechanisms of action, develop prognostic and diagnostic biomarkers, study off-target activity and model mechanisms of resistance [67, 68]. This can all be done for relatively small expense before embarking on expensive clinical trials [27].

Animal models may be of value in drug development for many major ophthalmic conditions, but it is important to be clear as to what aspects of pathogenesis or clinical manifestation the model is designed to recapitulate, and to recognise the differences that exist between even the best animal models and the human disease.

For example a pre-clinical animal model in glaucoma would ideally mimic the human disease by showing retinal ganglion cell and optic nerve damage brought about by chronic or transient ocular hypertension and would allow both frequent intra-ocular pressure measurements and easy visualisation of retinal neuronal damage [69]. Although such models exist, there are important considerations around anatomical differences between the species and pathophysiological differences between the disease processes. Anatomical differences between human and rodent eyes such as the small size of the rodent eye and differences in the lens and vitreous cavity are a significant limitation to the use (and interpretation) of rat and mouse glaucoma models. Non-human primate models have a number of pathophysiological advantages but are expensive and have limited availability [70]. In terms of disease initiation and ongoing pathogenesis, the key issue for all such models is the extent to which they occur ‘naturally’ or require ‘induction’ e.g. with injury by toxin or trauma [71]. A range of such glaucoma models exists across different species, but each has their limitations and no ideal animal model currently exits [69]. This potential pathophysiological discordance applies equally to a number of animal models for other disease processes, for example inducing CNV by laser disruption in a mouse to mimic age-related CNV in human macular degeneration [72]; or inducing uveitis with injection of lipopolysaccharide into the murine peritoneum to mimic acute anterior uveitis [73]. Newer iterations of these animal models, such as the numerous mouse models of CNV generation associated with a range of complement, chemokine or chemokine receptor abnormalities may more accurately mimic the human disease [72].

A further challenge is that some ocular diseases, such as TED have been strikingly resistant to modelling in animals. For a long time there was no robust animal model for TED, with none of the models being felt to exhibit the full spectrum of Grave’s disease or demonstrate a primary role for the Thyroid Stimulating Hormone Receptor (TSH-R) as an orbital target antigen [74]. A number of TSH-R induced murine models were attempted with either transfer of TSH-R-primed T cells to naïve syngeneic recipients, the use of a TSH-R fusion protein or genetic immunisation with a plasmid encoding the TSH-R to generate TSH-R-primed T cells [75]. Thyroiditis had been transferred to NOD (non obese diabetic) and BALB/c mice, but this was associated with only low titres of TSH-R antibodies (which were predominantly inhibitory rather than stimulatory) and no evidence of orbital disease in a significant proportion of the mice [76]. However, examination of the orbits in 17 of 25 of animals showed lymphocytic and mast cell infiltration, accumulation of adipose tissue, dissociation of muscle fibres and evidence of TSH-R immunoreactivity, whereas control mice showed no such eye pathology [76–79]. This was a predominantly Th2-mediated thyroiditis, with the extent of the orbit changes correlating with the extent of the Th2 response in the thyroid immune infiltrate. It has been observed, however, that different methods of TSH-R vaccination may lead to Th1 responses in which IFN-γ, rather than autoantibody, lead the immune response [75, 76]. More recently, Moshkelgosha et al. (2013) found that all 22 female BALB/c mice immunised with human TSH-R A-subunit plasmids by in vivo muscle electroporation gained clinical and histopathological features of TED, with evidence of asymmetric but bilateral enlarged extraocular muscles, proptosis and indications of orbital congestion, clinically and on in vivo MRI, as compared with those injected with control plasmids [80]. In addition, histopathology of orbital tissue demonstrated infiltration of CD3+ T lymphocytes, macrophages and mast cells, as well as glycosaminoglycan deposition, although no B lymphocytes. The histological findings were heterogeneous, with some mice manifesting predominantly extraocular abnormalities, with interstitial inflammatory infiltrate or otherwise adipogenesis with expansion of retro-ocular adipose tissue. Furthermore, all animals had high levels of TSH-R antibodies, predominantly with stimulatory function, which persisted up to 15 weeks after plasmid immunisation.

It is valuable to look outside of ophthalmology to see how animal models are advancing in other specialities. In oncology, xenograft based pre-clinical mouse models have predominated [67, 81]. It is well established that these models, whilst being cheap and easy to use, do not truly model human malignancies. Many drug candidates have shown strong anti-cancer activity in xenograft models but have very often gone on to fail in the later stages of clinical development [81]. In order to address these limitations, the focus has moved towards generating genetically engineered mouse models (GEMM) which mimic the spontaneous nature of tumours and closely recapitulate the human disease. These models have provided data about the mechanisms of tumour initiation, progression, maintenance and resistance mechanisms [82]. In order to validate particular GEMMs researchers have tried to retrospectively replicate clinical trial results in these models. This approach has been successful in two leading GEMMs: a Kras-driven non-small cell lung carcinoma model and a pancreatic ductal adenocarcinoma model [67]. This data provides further support for the use of GEMMs in modelling therapeutic response and in predicting the outcome of disease. A number of therapeutic agents that completed their pre-clinical development in GEMMs are currently in early stage clinical trials for the treatment of specific cancer types. The hope is that these advancements in pre-clinical development will be reflected in improved clinical success rates for these novel therapeutics.

#### Drug delivery-based advances

Whilst systemic drug delivery routes utilised in treating many diseases are often limited by systemic toxicity issues, the eye offers the tantalising opportunity to treat ocular disease directly in an isolated environment with potentially no systemic side effects. To realise this advantage drug delivery systems have to overcome many ocular barriers that limit drug transport into the eye and lower the efficacy of many drugs [83]. The goal in ocular drug delivery is to develop novel, safe, and patient acceptable drug delivery technique(s) which can overcome these barriers and deliver active drug at the right dose to the correct sites.

Ocular drug delivery is generally divided into three categories: topical agents mainly targeting the anterior segment of the eye; intraocular/intravitreal agents which typically target the posterior segment; and systemic agents which can be used to treat both anterior and posterior segment diseases.

Topical eye drops are the most convenient and patient acceptable route of drug administration. This simple delivery system has been used for many centuries and still dominates the market today. Most conditions affecting the ocular surface and anterior chamber can be treated with topical formulations, however limitations include poor penetration across the cornea (<5 %) and low levels of accumulated drug due to rapid tear washout [32, 33, 84]. A focus of development has been around creating novel formulations of conventional topical solutions to address these two limitations and to improve patient adherence [33, 85]. Other approaches include the development of lipophilic pro-drugs of commonly used topical ocular therapies like timolol. This technique improves penetration of the drug and subsequently reduces systemic side-effects due to a reduced dose requirement [86].

Despite the ocular drug market being dominated by anterior segment disease therapies there has also been good progress in ocular drug delivery systems targeting posterior ocular diseases [84, 87]. Since the advent of anti-VEGF therapies, the intravitreal injection route has been widely used to deliver therapeutic entities to the retina, and is now the standard method of local delivery to the posterior segment. Despite lowering the risk of systemic exposure to drug their requirement for repeated invasive administration is associated with a significant cumulative risk of complications including retinal detachment, endophthalmitis and increased ocular pressure [88]. To overcome these limitations and in order to expand the repertoire of drugs that can be used to treat posterior segment disease a number of novel drug-delivery systems have been developed.

##### Ocular implants

These are designed with the aim of providing sustained release of drug over several months to years. Many novel patented systems are in development [84] and results to date demonstrate an improvement in bioavailability combined with superior patient safety and fewer side effects due to a reduction in injection frequency. On Demand Therapeutics have developed an intravitreal implant that contains sealed reservoirs of drugs which can be released in a non-invasive manner using as a slit-lamp directed laser [89]. Neurotech have taken an alternative approach and developed an Encapsulated Cell Technology (ECT) implant. ECT implants contain genetically engineered cell lines that continuously deliver therapeutic proteins directly into the vitreous for up to 2 years [90]. Phase II trials for implants containing cells secreting factors that treat pathological angiogenesis are currently underway in dry AMD (NCT00447954) and in both early stage retinitis pigmentosa (NCT00447980) and in late stage retinitis pigmentosa (NCT00447993) [91]. There are a number of other drug delivery systems including drug-eluting punctal plugs, drug-eluting contact lenses and anterior chamber implants [92, 93].

##### Ocular iontophoresis

This is a non-invasive technique that uses a low electric current to improve the uptake of ionic drug into tissue, particularly across the cornea and sclera. Fast and safe delivery of high dose ophthalmic drug concentrations using transcorneal iontophoresis has previously been demonstrated for antibacterial, antiviral, and antifungal drugs, steroids, antimetabolites, RNA and DNA molecules with encouraging results [32, 87].

With its ease of application, a reduction in systemic side effects and increased drug penetration iontophoresis is an attractive area for commercialisation. EyeGate have developed an advanced ocular iontophoresis device and have completed a Phase III study of their lead drug candidate EPG-437 (dexamethasone phosphate formulated for iontophoresis) in non-infectious Anterior Uveitis [94]. EPG-437 produced the same efficacy outcomes over a four week period compared to the current standard of care (prednisolone eye drops) while eliminating the need of applying up to 8 eye drops a day. This was achieved with a significantly lower incidence of increased intraocular pressure. This clinical data shows promising signs of efficacy, may address compliance issues and may lead to a more predictable clinical response in treating severe uveitis [84, 95].

The development of non-invasive delivery techniques has the potential to revolutionise ocular drug discovery. However, for this to be realised ocular drug delivery systems must be considered and optimised throughout the drug discovery process. Business models involving non-exclusive partnering of platform drug delivery systems with companies developing therapeutic agents is a model that may lead to success.

#### Clinical trials and patient stratification

In order to improve the current success rate of a novel therapeutic agent in Phase II trials [96] the drug discovery industry has started to move away from classifying disease cohorts based on their clinical presentation and moved towards classifying based on the underlying pathophysiology of an individual’s disease. This has led to the concept of personalised medicine in which therapies are targeted to patient sub-populations based on their specific disease type [97]. The concept is based on the observation that in some clinical trials only subgroups of patients responded to treatment [98, 99]. By analyzing responders, it is possible to develop stratification or predictive biomarkers that can prospectively predict which patient groups will respond to a given treatment. The field of oncology has led the way and with the highly heterogeneous nature of the disease it is an ideal platform for the development of stratification biomarkers [97]. Recent successes include the approval of Zelboraf (Vemurafenib) as a monotherapy alongside a companion genetic test for BRAF mutations for the treatment of adult patients with BRAF V600E mutation-positive unresectable or metastatic melanoma [100]. Vemurafenib was developed as a specific inhibitor of the mutant V600E BRAF and showed a 74 % relative reduction in death or disease progression compared to standard treatment in Phase III trials [101]. Current research suggests that stratification by biomarkers used in clinical trials reduces the size and length of the clinical study. This results in significant development cost savings and an increased chance of clinical trial success [102].

Within ophthalmology there are likely to be a number of conditions that have been grouped together due to superficial clinical resemblance, but which arise from different aetiological processes, and so may require different therapeutic strategies. Denniston and Dick have argued how this applies to the challenging area of ocular inflammatory disease, where many forms of uveitis are ‘lumped together’ in the same clinical trial, but may arise from different derangements of the immune system, may require different outcome measures and may show different responses to any one drug [38]. This ‘lumping together’ arises for a number of reasons including poor understanding of pathogenesis, lack clear diagnostic markers, and poorly defined disease phenotypes. Additionally, at the pragmatic level, their individually rarity has pushed investigators to increase the size of trial by broadening the uveitis types included, for example to ‘all non-infectious posterior segment-involving uveitis’. Under such circumstances there is a high risk of failing to identify a successful treatment due to the low “signal-noise” ratio [38]. In contrast adopting a similar biomarker-based patient stratification approach as used in oncology trials, might enable more successful, smaller trials with significant cost-savings [102].

## Conclusion

Drug discovery is an essential part of ophthalmic care, achieving ever more effective and safe treatments that appropriately target the site of disease and are acceptable to the patient. Whilst some large groups of patients are now benefiting from major breakthroughs such as anti-VEGF therapies in wet AMD, diabetic macular oedema and retinal vein-occlusive diseases, there is still a huge burden of both ‘untreatable’ eye disease or ‘under-treated’ disease where effective treatments are not yet available or are poorly tolerated.

In this review we have considered how past success has given way to increasing challenges within the drug discovery industry. We have discussed what attempts are being made to increase the productivity of drug development, including the refocusing on the study of the basic biology of the disease. We have considered what ophthalmic drug discovery can learn from the sector in general and have discussed strategies which seek to overcome the present limitations such as the advances in the understanding of the pathogenesis of disease, improvements in animal models of human disease, approaches to ophthalmic drug delivery and attempts at patient stratification within clinical trials. Encouragingly the most recent data from the FDA suggests that we may now be experiencing an upturn in the number of new products being licensed, with a total of 41 approvals for NME and biological products in 2014. The future holds great promise for safe and effective therapies for ocular disease but this depends on ongoing investment in ophthalmic drug development covering the whole translational spectrum from ‘bench to bedside’.
